# Association of *TERT* polymorphisms and risk of coronary heart disease in a Chinese Han population

**DOI:** 10.18632/oncotarget.18727

**Published:** 2017-06-28

**Authors:** Hongmei Han, Jianxia Zhang, Jianghong Hou, Haibo Wang, Jianpeng Zheng, Huan Wang, Zhong Zhong, Yijin Wang, Xiaoni Wang, Bei Yang, Lei Wang, Dangjun Quan, Junnong Li

**Affiliations:** ^1^ Department of Cardiovascular Medicine, Weinan Central Hospital, Weinan 714000, Shaanxi, China

**Keywords:** coronary heart disease (CHD), *TERT*, telomere, single nucleotide polymorphisms (SNP), case-control study

## Abstract

Genome-wide association studies have identified that *TERT* gene was associated with telomere length and age-related diseases. However, little study directly focused on the association between *TERT* gene polymorphisms and risk of coronary heart disease (CHD). We conducted a case-control study to examine the effect of *TERT* polymorphisms on CHD risk among 596 CHD patients and 603 healthy controls from China. Five significant single nucleotide polymorphisms (SNP) in *TERT* were selected and genotyped using Sequenom Mass-ARRAY technology. Odds ratios (OR) and 95% confidence intervals (CIs) were calculated using unconditional logistic regression adjusting for age and gender. Allelic model analysis revealed that for *TERT* rs10069690, allele frequency distributions differed between cases and controls (OR= 1.267, 95%CI = 1.018-1.576; *p* = 0.034). Genotypic model analysis revealed that genotype frequency distributions of rs10069690 differed between cases and controls after adjusted by age and sex (TC vs. CC: adjusted OR = 1.352, 95% CI = 1.007-1.815; *p* = 0.045). Genetic model analysis revealed that rs10069690 was associated with an increased risk of CHD under co-dominant, dominant, over-dominant and log-additive models. After adjustments, it remained significant under over-dominant model (adjusted OR = 1.35, 95% CI = 1.01-1.81; *p* = 0.044). Our results shed new light on the association between telomere-related gene *TERT* polymorphisms and CHD susceptibility in a Chinese Han population.

## INTRODUCTION

Coronary heart disease (CHD), including myocardial infarction, angina pectoris and arteriosclerosis of the coronary arteries, is the leading cause of disability and mortality world-wide [1–3]. Previous studies have revealed that CHD is a complex polygenic disease, and genetic factors are crucial to an individual’s susceptibility to CHD. [4]. To date, genome-wide association studies (GWAS) have identified more than 40 common variants associated with the risk of CHD [5, 6]. However, this is not enough to explain the etiology of CHD. In recent years, researchers were still working on looking for novel susceptibility locus for CHD and replicating significant single nucleotide polymorphisms (SNP) in different populations.

Telomeres are located at the ends of chromosomes, which consist of tandem (TTAGGG)_n_ nucleotide repeats and some binding proteins [7]. The average telomere length is about 10-15 kb in human somatic cells, and shorten in most cells with aging [8]. Telomere plays a significant role in maintaining the stability and integrity of the genome [9]. Telomerase reverse transcriptase is required to keep the maintenance of telomere [10]. Loss of telomere function and infinite proliferation leads to genomic instability and chromosomal abnormalities, which may promote carcinogenesis [11].

The *TERT* gene at 5p15.33 encodes the catalytic subunit of telomerase reverse transcriptase, which is an important component of telomerase. GWAS studies have identified that *TERT* gene was associated with telomere length and age-related diseases [12]. However, little study directly focused on the association between *TERT* gene polymorphisms and CHD risk. We performed a case-control study to analyze the association between five SNPs in *TERT* and the risk of CHD in a Chinese Han population.

## RESULTS

A total of 596 CHD cases (376 men and 220 women; mean age, 61.44 ±11.16 years) and 603 controls (469 men and 134 women; mean age, 48.24 ±13.05 years) were included in the study. The clinical characteristics of the cases and controls are shown in Table 1. There were significant differences in the age and gender distributions between the case and control groups (*p* < 0.05). Multivariate analyses were adjusted for age and sex.

**Table 1:** Characteristics of cases and controls included in this study

Variables	Case (N=596)	Control (N=603)	*p*-value
Sex, No.(%)			< 0.001^a^
Male	376	469	
Female	220	134	
Mean age ±SD	61.44 ±11.16	48.24 ±13.05	< 0.001^b^

^a^The *p* value was calculated from Pearson’s chi-square tests.

^b^The *p* value was calculated by Welch’s t tests.

SD, standard deviation.

The minor allele frequencies (MAFs) of the analyzed SNPs in the case and control groups are shown in Table 2. All SNPs were in Hardy-Weinberg equilibrium (HWE) in the controls (*p* > 0.05). The MAFs of the SNPs in the control group were similar to those reported for the HapMap Asian population. Using chi-square tests, we determined that rs10069690 was associated with a 1.267-fold increase in the risk of CHD (95%CI = 1.018-1.576; *p* = 0.034). No significant associations were detected between the other SNPs and CHD risk.

**Table 2:** Allele frequencies in cases and controls and odds ratio estimates for CHD

SNP ID	Band	Position	Gene	Alleles A^a^/B	MAF		HWE *p*-value	ORs(95%CI)	*p*-value
					Case	Control			
rs2075786	5p15.33	1266310	*TERT*	G/A	0.163	0.162	0.879	1.003(0.806-1.248)	0.981
rs10069690	5p15.33	1279790	*TERT*	T/C	0.180	0.147	0.190	1.267(1.018-1.576)	0.034*
rs2242652	5p15.33	1280028	*TERT*	A/G	0.171	0.163	0.549	1.064(0.858-1.319)	0.571
rs2853677	5p15.33	1287194	*TERT*	G/A	0.377	0.366	0.793	1.048(0.888-1.237)	0.577
rs2853676	5p15.33	1288547	*TERT*	T/C	0.175	0.154	0.756	1.164(0.937-1.444)	0.169

SNP, single nucleotide polymorphism; MAF, minor allelic frequency; HWE, Hardy-Weinberg equilibrium; ORs, odds ratios; CI, confidence interval

^a^Minor allele; *p value ≤ 0.05 indicates statistical significance;

The genotype frequencies of the *TERT* polymorphisms are shown in Table 3. Compared to the CC genotype, the frequency of the TC genotype of rs10069690 polymorphism in the case group significantly differed from the controls (TC vs. CC: OR = 1.452, 95% CI = 1.122-1.879; *p* = 0.004), suggesting that rs10069690 increased the risk of CHD. It is remained significant after adjusted by age and sex (TC vs. CC: adjusted OR = 1.352, 95% CI = 1.007-1.815; *p* = 0.045).

**Table 3:** Genotypes frequencies of the SNPs and their associations with risk of CHD

SNP ID	Alleles A/B	Genotype	Genotype frequencies		Without adjustment		With adjustment	
			Case	Control	OR(95%CI)	*p*^a^	OR(95%CI)	*p*^b^
rs2075786	G/A	AA	419(70.3%)	411(70.3%)	1.00		1.00	
		GA	160(26.8%)	158(27.0%)	0.993(0.767-1.286)	0.960	0.925(0.688-1.243)	0.605
		GG	17(2.9%)	16(2.7%)	1.042(0.520-2.091)	0.907	0.972(0.431-2.191)	0.945
rs10069690	T/C	CC	396(66.4%)	436(73.4%)	1.00		1.00	
		TC	186(31.2%)	141(23.7%)	1.452(1.122-1.879)	0.004*	1.352(1.007-1.815)	0.045*
		TT	14(2.4%)	17(2.9%)	0.907(0.441-1.863)	0.790	1.020(0.452-2.301)	0.961
rs2242652	A/G	GG	404(67.8%)	425(70.5%)	1.00		1.00	
		AG	180(30.2%)	160(26.5%)	1.183(0.919-1.524)	0.192	1.088(0.817-1.453)	0.569
		AA	12(2.0%)	18(3.0%)	0.701(0.334-1.474)	0.349	0.693(0.297-1.616)	0.396
rs2853677	G/A	AA	225 (37.8%)	244 (40.5%)	1.00		1.00	
		GA	293 (49.2%)	277 (46.0%)	1.147(0.898-1.465)	0.271	1.163(0.880-1.535)	0.288
		GG	78 (13.0%)	82 (13.5%)	1.032(0.720-1.477)	0.865	1.259(0.834-1.902)	0.272
rs2853676	T/C	CC	405(68.0%)	429(71.3%)	1.00		1.00	
		TT	173(29.0%)	160(26.6%)	1.145(0.888-1.477)	0.296	1.153(0.862-1.544)	0.337
		TC	18(3.0%)	13(2.1%)	1.467(0.710-3.032)	0.301	1.896(0.838-4.290)	0.124

SNP, single nucleotide polymorphism; OR, odds ratio; 95%CI, 95% confidence interval.

^a^*P* values were calculated from unconditional logistic regression analysis.

^b^*P* values were calculated by unconditional logistic regression analysis with adjustments for age and gender.

*P≤0.05 indicates statistical significance.

We assumed that the minor allele of each SNP was a risk factor compared to the wild-type allele. Five genetic models (co-dominant, dominant, recessive, over-dominant and log-additive) were applied to analyze the associations between the SNPs and CHD risk using an unconditional logistic regression analysis with adjustments for age and gender (Table 4). We found that rs10069690 was associated with an increased risk of CHD under co-dominant, dominant, over-dominant and log-additive models. After adjustments, it remained significant under over-dominant model (adjusted OR = 1.35, 95% CI = 1.01-1.81; *p* = 0.044).

**Table 4:** Association between rs10069690 and risk of CHD in multiple inheritance models (adjusted by age and gender)

Model	Genotype	Group=case	Group=control	Without adjustment				With adjustment			
				OR (95% CI)	*p*	AIC	BIC	OR (95% CI)	*p*	AIC	BIC
Co-dominant	C/C	396 (66.4%)	436 (73.4%)	1	0.015*	1647.3	1662.5	1	0.13	1356.2	1381.6
	C/T	186 (31.2%)	141 (23.7%)	1.45 (1.12-1.88)				1.35 (1.01-1.82)			
	T/T	14 (2.4%)	17 (2.9%)	0.91 (0.44-1.86)				1.02 (0.45-2.30)			
Dominant	C/C	396 (66.4%)	436 (73.4%)	1	0.0088*	1646.8	1657	1	0.056	1354.7	1375
	C/T-T/T	200 (33.6%)	158 (26.6%)	1.39 (1.09-1.79)				1.32 (0.99-1.75)			
Recessive	C/C-C/T	582 (97.7%)	577 (97.1%)	1	0.58	1653.4	1663.5	1	0.88	1358.3	1378.6
	T/T	14 (2.4%)	17 (2.9%)	0.82 (0.40-1.67)				0.94 (0.42-2.11)			
Over-dominant	C/C-T/T	410 (68.8%)	453 (76.3%)	1	0.0039*	1645.3	1655.5	1	0.044*	1354.2	1374.5
	C/T	186 (31.2%)	141 (23.7%)	1.46 (1.13-1.88)				1.35 (1.01-1.81)			
Log-additive	---	---	---	1.27 (1.02-1.58)	0.033*	1649.1	1659.3	1.23 (0.96-1.58)	0.1	1355.7	1376

ORs, odds ratios; CI, confidence interval; AIC, Akaike’s Information criterion; BIC, Bayesian Information criterion.

*p value ≤0.05 indicates statistical significance.

**Figure 1:**
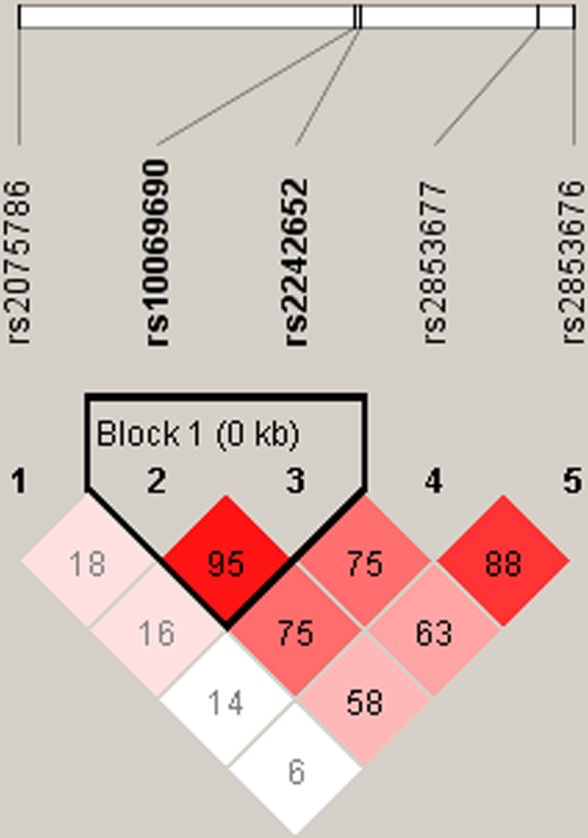
D’ linkage map for the five SNPs in *TERT*

We further characterized the SNPs in *TERT* SNPs using LD and haplotype analyses. One block including rs10069690 and rs2242652 was detected (Figure 1). However, we didn’t found any significant result in the haplotype analysis (Table 5).

**Table 5:** *TERT* haplotype frequencies and the association with the CHD risk

Haplotype	rs10069690	rs2242652	Freq-case	Freq-control	Without adjustment		With adjustment	
					OR (95% CI)	*p*	OR (95% CI)	*p*
1	C	G	0.818	0.832	1	---	1	---
2	T	A	0.169	0.146	1.17 (0.94 - 1.46)	0.16	1.11 (0.86 - 1.44)	0.41
rare	*	*	0.013	0.022	0.61 (0.32 - 1.16)	0.13	0.72 (0.35 - 1.46)	0.36

Freq, frequency; ORs, odds ratios; CI, confidence interval.

## DISCUSSION

In this study, we investigated the associations between five selected *TERT* SNPs and risk of CHD in a Chinese Han population. We found that rs10069690 is associated with an increased risk of CHD. Our results suggest that the polymorphisms of *TERT* may play an important role in the risk of CHD in a Chinese Han population.

*TERT* gene encodes the telomerase reverse transcriptase, which is really important to keep the balance of telomere length [13]. It has been demonstrated that mean leucocyte telomere length is a predictor of the development of CHD, and differences in biological ageing might contribute to the risk and variability in age of onset of CHD [14]. In the present study, we further demonstrated that *TERT* gene polymorphisms were associated with CHD risk, which shed new light on the association between telomere length and CHD risk. Actually, *TERT* has been associated with many types of cancer, including lung cancer, urinary bladder, prostate and cervix cancer, and so on [15]. We suggested that *TERT* gene may have relationship with cancers and disease by influencing the balancing the telomere length; however, the mechanistic details have not yet been elucidated.

Previous association studies have found many SNPs associated with CHD risk in different populations. Samani et al. found that rs1333049, rs3008621, rs599839, rs501120, rs2943634 and rs6922269 were associated with CHD risk in populations of European ancestry [16]. Tang, Wang, Lu et al. have replicated these locus and explore novel loci in the Chinese population, including rs6903956, rs1842896, rs2123536, rs7136259 and rs9268402 [17–19]. In the present study, we selected five SNPs in *TERT* gene, including rs2075786, rs10069690, rs2242652, rs2853677 and rs2853676. Rs2075786 was found to be associated with paranoid schizophrenia [20] and colorectal cancer risk. However, the results were inconsistent [21, 22]. In our study, we didn’t observe any association between rs2075786 and CHD risk. Rs10069690 was found to be associated with breast cancer [23], hepatocellular carcinoma and tumor metastasis [24]. We are the first to report that rs10069690 was associated with increased risk of CHD, which need to be confirmed in further studies. For the remaining three SNPs rs2242652, rs2853677 and rs2853676, Nan et al. reported that rs2242652 and rs2853676 were associated with risk of melanoma [25]. Recently, Li et al. reported that rs2853677 modulates Snail1 binding to the *TERT* enhancer and affects lung adenocarcinoma susceptibility [26]. However, little information is found about the association between these SNPs and CHD risk. We didn’t detect any association between rs2242652, rs2853677 and rs2853676 and CHD risk, either.

The present study has several potential limitations. First, the sample size is relatively small, and the participants included only Chinese population lived in Shaanxi Province. Second, CHD is a multifactoral disease that contains several complex genetic and environmental factors. We could not completely eliminate the potential influences of environmental factors on the results. Therefore, the interaction of genetic and environmental factors in the development of CHD need be confirmed in further studies with a larger and multifarious sample.

In sum, our results indicate that *TERT* rs10069690 is associated with an increased risk of CHD, which may has the potentially to serve as prognostic biomarker for CHD among the Chinese Han population. Further study will focus on validating our findings with a larger sample and on determining the functional role of these SNPs.

## MATERIALS AND METHODS

### Ethics statement

We strictly obeyed the World Medical Association Declaration of Helsinki when using human tissue and signing the study protocol with subjects, which was approved by the Ethical Committee of Weinan Central Hospital. Each participant provided written, informed consent.

### Subjects

All participants in our study were Han Chinese. A total of 596 CHD patients and 603 healthy controls were consecutively recruited between January 2014 and May 2016 in the Weinan Central Hospital in Weinan city, China. Patients were diagnosed with CHD using standard coronary angiography, which revealed ≥ 70% stenosis of the main branch of a coronary artery or aortic stenosis ≥ 50%. Subjects with myocardial infarction, stable angina and unstable angina were classified as CHD subjects. There were no age, sex or disease-stage classification restrictions when enrolling the case group. Controls were healthy people receiving physical examinations in other clinical departments of Weinan Central Hospital. Healthy controls have no congenital heart disease, familial hypercholesterolemia, end-stage renal disease and known vasculitides, which could have affected our study results. Peripheral blood was collected from both cases and controls for DNA extraction.

### SNP selection and genotyping

Candidate SNPs in the *TERT* gene were selected from previous publications that associated polymorphisms with telomere length [27, 28]. SNPs with minor allele frequencies (MAF) > 5% in the HapMap CHB population were selected. We validated five SNPs in *TERT*. The GoldMag-Mini Purification Kit (GoldMag Co. Ltd. Xian city, China) was used to extract genomic DNA from whole blood samples. DNA concentration was measured using a DU530 UV/VIS spectrophotometer (Beckman Instruments, Fullerton, CA, USA). Using MassARRAY Assay Design 3.0 software (Sequenom, San Diego, CA, USA), we designed a multiplexed SNP MassEXTENDED assay [29]. SNPs were genotyped using the standard protocol recommended by the MassARRAY RS1000 (Sequenom) manufacturer and data were analyzed using Typer 4.0 Software (Sequenom). Primers used for this study were listed in Table 6.

**Table 6:** Primers used for this study

SNP_ID	First PCR Primer	Second PCR Primer	UEP SEQ
rs2075786	ACGTTGGATGCAGGTTACACACGTGGTGAG	ACGTTGGATGCGCCACTCTTGACTTTCCAA	ggCAAAGAGCAGCAGGAGCC
rs10069690	ACGTTGGATGCCTGTGGCTGCGGTGGCTG	ACGTTGGATGATGTGTGTTGCACACGGGAT	GGGATCCTCATGCCA
rs2242652	ACGTTGGATGACAGCAGGACACGGATCCAG	ACGTTGGATGAGGCTCTGAGGACCACAAGA	gtcgGAGGACCACAAGAAGCAGC
rs2853677	ACGTTGGATGATCCAGTCTGACAGTCGTTG	ACGTTGGATGGCAAGTGGAGAATCAGAGTG	gggtAATCAGAGTGCACCAG
rs2853676	ACGTTGGATGTGTCTCCTGCTCTGAGACC	ACGTTGGATGCAAAACTAAGACCCAAGAGG	agatGGAAGTCTGACGAAGGC

UEP SEQ, unextended mini-sequencing primer.

### Statistical analysis

We used Microsoft Excel and SPSS 17.0 (SPSS, Chicago, IL) statistical packages to perform statistical analyses. All *p*-values were two-sided and *p*<0.05 was considered statistically significant. A *t* test and Chi-square test were performed to compare sex and age differences between cases and controls. Fisher’s exact test was applied to each SNP in the controls to test for departure from Hardy–Weinberg Equilibrium (HWE). Odds ratios (ORs) and 95% confidence intervals (CIs) for the allele and genotype frequencies were calculated using Pearson Chi-square test adjusted by age and sex [30]. Five models (co-dominant, dominant, recessive, over-dominant and log-additive) were used to assess the association between each genotype and the risk of CHD. The effects of the polymorphisms on the risk of CHD were expressed as ORs with 95% CIs, which were calculated using unconditional logistic regression analysis after adjusting for age and gender [31]. Akaike's information criterion (AIC) and Bayesian information criterion (BIC) were applied to choose the best-fit model for each SNP. Finally, linkage disequilibrium (LD) patterns and haplotypes were evaluated using the Haploview software package (version 4.2) [32].
